# Validation of the Implementation Climate Scale (ICS) in substance use disorder treatment organizations

**DOI:** 10.1186/s13011-019-0222-5

**Published:** 2019-08-23

**Authors:** Mark G. Ehrhart, Elisa M. Torres, Joyce Hwang, Marisa Sklar, Gregory A. Aarons

**Affiliations:** 1 Department of Psychology, University of Central Florida, Orlando, FL USA; 2 Department of Psychology, George Mason University, Fairfax, VA USA; 3 Department of Psychology, San Diego State University, San Diego, CA USA; 4 Department of Psychiatry, University of California, La Jolla, San Diego, CA USA; 5 Child and Adolescent Services Research Center, San Diego, CA USA

**Keywords:** Implementation climate, Leadership, Substance use disorder treatment, Addictions, Confirmatory factor analysis, Organizational readiness

## Abstract

**Background:**

One critical factor in the implementation of evidence-based practice (EBP) in substance use disorder treatment organizations is an inner organizational context that clearly supports implementation efforts. The Implementation Climate Scale (ICS) has been developed to allow researchers and organizations to assess climate for EBP implementation in health and allied health service organizations. The ICS consists of 18 items and measures six dimensions of implementation climate: focus on EBP, educational support for EBP, recognition for EBP, rewards for EBP, selection for EBP, and selection for openness. The ICS was initially developed in a mental health context; thus, the goal of this study was to provide initial validation of the ICS in substance use disorder (SUD) treatment settings.

**Methods:**

Confirmatory factor analysis (CFA) was used to assess the psychometric functioning of the ICS using survey data from 326 providers in 65 teams in SUD treatment programs. Cronbach’s alpha was examined to assess internal consistency of the ICS, and individual and team level construct-based validity was examined by comparing its correlations with service climate, molar climate, and organizational change.

**Results:**

We found evidence for the reliability, factor structure, and validity of the ICS in SUD services. The psychometric functioning of the ICS in SUD treatment settings was comparable to that found in mental health contexts.

**Conclusions:**

The ICS is a brief and pragmatic tool for researchers to better understand a critical antecedent for implementation effectiveness in SUD treatment and for organizational leaders in SUD treatment organizations to evaluate the extent to which providers perceive that their organization supports EBP implementation.

## Background

Implementation of evidence-based practices (EBPs) is critical for improving care in the public health and allied health sectors such as substance use disorder (SUD) treatment settings. Although EBPs are known to researchers as efficacious practices supported by research evidence, there is still a discrepancy between the knowledge that EBPs are available and the usage of EBPs by providers (i.e., clinicians) with their clients [1–3]. This discrepancy is apparent in SUD services, and can be partly attributed to the addiction field’s historical roots of development outside of mainstream health care [4–6].

Overall, implementation of EBPs for SUD has received less attention than for mental health [7–13]. The unique development of SUD treatment systems, which evolved separately from mainstream health care and from mental health treatment systems has contributed to this science to practice gap in SUD services [10, 14]. Through the mid-1900s, individuals with SUD were socially stigmatized and were refused treatment from most practitioners and hospitals [10]. This resulted in an emergence of alternative treatment offered primarily through “compassionate peers who were themselves in recovery” ([10], p. 26). As time progressed, a chronic illness/disease model was accepted for alcohol use disorders, and subsequently for other drug use disorders, though SUD treatment remained segregated from medical and mental health services. A lack of centralized, federal, policies governing the SUD system resulted in each state designing individual public SUD health care systems with varying organizational and financing structures [15]. Differences emerged between SUD and mental health with regard to insurance coverage, such that clients may have coverage that provides mental health services but excluded SUD treatment [16, 17]. SUD treatment providers, programs, and systems grew more strongly loyal to particular treatment models, often with limited scientific evidence for efficacy [18, 19]. Specifically, SUD treatment practices continued to be influenced by the experiences of people in recovery, primarily through Alcoholics Anonymous and related 12-step programs [10].

At present, the literature reflects a general openness of SUD professionals to learning new practices [10]. Additionally, federal and state initiatives have made efforts to advance SUD EBP implementation [20]. For example, the National Institute on Drug Abuse (NIDA) Blue Ribbon Task Force encouraged an increase in funded studies examining substance use EBPs and their dissemination, implementation, and sustainment in real-world settings [21]. Within SUD health care systems, instability and closure of treatment programs is common and further warrants investigation of EBP implementation in such systems [22].

Within implementation research, there has been an increasing interest in the organizational context within treatment agencies and how the environment within which providers work impacts the successful uptake and implementation of EBPs [23]. As a guide for the factors that may affect implementation in public service sectors, Aarons and colleagues [24] developed the Exploration, Preparation, Implementation, Sustainment (EPIS) multilevel implementation framework, which characterizes four phases of the implementation process and categorizes implementation factors into outer (system) and inner (organizational) context. Within SUD treatment settings, researchers have identified several outer system factors which impact successful EBP implementation, including interagency collaborations [25] and network connectedness with non-criminal justice agencies [26]. Moreover, several inner organizational factors have also been identified as playing a pivotal role in whether EBPs are successfully implemented, such as leadership [26], counselor attitudes toward EBP [27, 28], and perceptions of organizational readiness for change [29–31].

The focus of the present study is on one particular indicator of the inner organizational context for EBP implementation: organizational climate. In recent years, there has been an increasing interest in examining the influence of organizational climate on the EBP implementation process (e.g., [32–34]). Organizational climate has been defined as “the shared meaning organizational members attach to the events, policies, practices, and procedures they experience and the behaviors they see being rewarded, supported, and expected” ([35], p. 69). Research on organizational climate has been categorized by two primary approaches: those addressing molar climate and those addressing a more specific or focused climate [35]. Generic or molar climates capture the overall organizational environment and employees’ experiences in an organization as a whole. In contrast, focused climates capture factors that are relevant to attaining specific strategic outcomes, such as customer service [36] and safety [37].

Of the SUD treatment research targeting the role of the organizational context in EBP implementation, generic or molar organizational climate has typically been the focus, measured as part of the Organizational Readiness for Change (ORC) assessment [31]. The ORC is posited as a comprehensive appraisal of organizational functioning which includes four domains: 1) motivation for change, 2) resources, 3) staff attributes, and 4) organizational climate [38]. Within the organizational climate domain of the ORC are six scales: clarity of mission and goals, staff cohesiveness, staff autonomy, openness of communication, stress, and openness to change. In line with molar climate measures, these scales capture general organizational functioning and are not specific to a specific strategic imperative (such as implementation). A recent review found the ORC was used in multiple studies examining the influence of organizational readiness for change on EBP implementation in SUD treatment programs; however, the application of ORC findings in the process of implementing quality improvement initiatives remains unclear [39].

Although much of the current research examining the impact of organizational climate on the implementation of EBPs has focused on molar climate [23, 29, 40, 41], researchers are now investigating the role of specific, focused climates, including the organization’s climate for EBP implementation [33, 42–44]. Strategic climates develop depending on how employees gauge management’s interpretation of the climate, and observing the degree to which leaders expect, support, and reward the use of certain targeted clinical and service strategies such as EBPs. Strategic EBP implementation climate develops when the organization and its leaders communicate and demonstrate their values regarding the importance of implementing EBPs. In organizations where EBP implementation climate is high, employees perceive that the organization supports the implementation of EBPs. In turn, employees also focus their attention on EBP implementation, and model these perceived organizational values. The presence of a strong EBP implementation climate should ultimately result in enhanced EBP specific outcomes [33, 42, 43, 45].

Building on foundational research on implementation climate by Klein, Conn, and Sorra [46] and Schein’s work on climate/culture embedding mechanisms [47], Ehrhart, Aarons, and Farahnak [43] defined an EBP implementation climate as “employees’ shared perceptions of the importance of EBP implementation within the organization” (p. 2). To measure the EBP implementation climate, they drew from past literature on implementation climate and other focused climates as well as subject matter expert input to develop the Implementation Climate Scale (ICS). The ICS identifies the following six factors that contribute to successful implementation climate: 1) the team/agency focus on EBP, 2) providing education support for EBP, 3) recognizing staff for utilizing EBP, 4) providing rewards to staff for employing EBP, 5) selecting staff who have prior EBP experience, and 6) selecting staff for general openness. Research on the ICS has shown that it is positively related with a variety of implementation-related outcomes [48–50]. Although this measure has been validated in mental health [43] and child welfare [51] settings, it has not been validated in SUD settings.

The purpose of this study was to examine the psychometric characteristics of the ICS in a sample of SUD service organizations. Although there are limited studies to date on implementation of EBPs in SUD as compared to mental health services, research on mental health EBP implementation can serve a useful guide [20]. As such, we hypothesized that the psychometric properties and factor structure of the ICS in SUD settings would be similar to what has been found in past research in examining the ICS in mental health services. Aligned with previous validation studies of the ICS [43], we evaluated construct-based validity with similar and relevant constructs to EBP implementation climate. We hypothesized that the ICS would have moderate positive correlations with another focused climate, service climate, which evaluates the organization’s emphasis on providing high quality services. Organizations that emphasize high quality service are likely also to place an emphasis on EBP implementation, but the distinct focus of each type of climate should mean that the measures are not completely overlapping. We hypothesized a weak positive correlation between the ICS and general molar climate, which is related to aspects of generally effective work units. Teams that are generally well-functioning may also be more likely to place an emphasis on EBP implementation, but the measures should be distinct because the content of molar climate is not implementation specific. We hypothesized a weak positive correlation between the ICS and planned organizational change, because even though organizations that place a strong emphasis on EBP implementation are likely to be undergoing change, the measure includes aspects of innovation and flexibility that are not central to EBP implementation. A weak negative correlation with uncertainty of organizational change was also hypothesized. Because teams with a strong implementation climate would have a structure and processes for successful implementation, we expect less uncertainty about change in such environments.

## Method

### Sample

Our sample consisted of 326 SUD service providers employed in three publicly funded, non-profit SUD organizations in California and New York. Of the 363 eligible providers, 327 (90.1%) participated in the survey. Of the 327 participants, one participant was not included in the analytic sample due to missing data for the entire ICS measure, resulting in a final analytic sample of 326. Of the 326 participants, 166 (50.9%) were from one organization (all in CA), another 107 (32.8%) from a second organization (54 in CA and 53 in NY), and 53 (16.3%) from a third organization (all in CA). Providers were organized into 65 teams (i.e., providers who report to the same supervisor), with an average team size of 5.02 (SD = 3.1; range = 1–13). Consistent with other research in SUD service settings [52], the sample was predominately female (62.9%) with an average age of 46.49 years (SD = 11.61, range 21–71). The racial distribution of the sample was 59.7% ‘Caucasian’, 18.9% ‘African-American’, 1.3% ‘Native-American’, 2.8% ‘Asian-American or Pacific Islander’, and 17.3% ‘Other’, with 28.5% of participants identifying as ‘Hispanic/Latino.’ The majority of participants completed at least some college education (90.5%), and 9.5%% indicating they had less than a college education. Additional demographic characteristics can be found in Table 1.

**Table 1:** Demographic characteristics for participant sample

Characteristics	Values
Race	
Caucasian	59.7% (190)
African-American	18.9% (60)
Asian-American or Pacific Islander	2.8% (9)
Native American	.1% (4)
“Other”	17.3% (55)
Ethnicity	
Hispanic	28.5% (93)
Non-Hispanic	71.5% (233)
Education	
No college	9.5% (30)
Some college	32% (101)
College degree	23.4% (74)
Some graduate work	5.7% (18)
Master’s degree	27.5% (87)
Ph.D. or M.D.	1.9% (6)
Gender	
Female	62.9% (205)
Male	37.1% (121)
Position	
Intern/trainee	20.5% (66)
Licensed provider	54% (174)
Neither	25.5% (82)
Primary discipline	
Drug/Alcohol Counseling	69.7% (219)
Marriage & family therapy	11.1% (35)
Psychology	8% (25)
Social Work	8% (25)
Other	3.2% (10)
Age	
Mean (*SD*)	46.5 (*11.6*)
Tenure with agency (years)	
Mean (*SD*)	3.7 (*3.7*)
Tenure in SUDT (years)	
Mean (*SD*)	7.0 (*6.1*)

Note: Due to missing data, values may not add up to total N of 326

### Procedure

Prior to recruitment, initial contact with agency executives was made to gain approval to proceed and to verify that the organization had recent, if not current, experience implementing an EBP. Upon approval, supervisors were then contacted via email to schedule a call presenting the study to their staff. During those calls, the research team provided further information about the definition of, and ensured staff familiarity with, EBP.

Data were collected via online (*n* = 222) and paper-and-pencil (*n* = 104) surveys depending on feasibility, and the survey took approximately 20–30 min to complete. A comparison of the means on the ICS subscales revealed no significant differences in the ICS measure based on the method of survey completion (online versus paper-and-pencil). This study was approved by the appropriate Institutional Review Boards and participants provided informed consent prior to survey administration, and received a $15 gift certificate for their participation. For online surveys each participant was emailed a unique password and username, in addition to the link to the survey. For in-person data collection the research team reserved an hour during a regularly occurring team meeting. Participants were ensured that their responses to the survey would be completely confidential and individually identifiable data would not be provided back to their supervisors. If participants were not able to complete the survey in-person and collecting data online was not practical, surveys were left or mailed to the participating agencies and returned to the research team via mail.

### Measures

#### Implementation climate

Implementation climate was measured using the Implementation Climate Scale (ICS; 43). Providers reported about their team’s (i.e., their work group’s) implementation climate. Specifically, the referent for the respondent was the team, which was determined based on the service provider’s direct supervisor. The ICS is comprised of 18 items with and respondents are asked to respond regarding the degree to which they agree with each statement using response anchors from 0 (‘not at all’) to 4 (‘to a very great extent’). Items fall into six subscales: 1) Focus on EBP (*α* = .90), 2) Educational support for EBP (*α* = .84), 3) Recognition for EBP (*α* = .78), 4) Rewards for EBP (*α* = .81), 5) Selection for EBP (*α* = .89), and 6) Selection for openness (*α* = .85). The mean of the subscales was computed to create the ICS score (*α* = .90). The complete ICS measure including scoring instructions can be found in the “additional files” accompanying the original measurement development study [43]; https://implementationscience.biomedcentral.com/articles/10.1186/s13012-014-0157-1.

#### Service climate

Service climate refers to employees’ perceptions of practices, procedures, and behaviors that are expected, rewarded, and supported with regard to customer service and customer service quality. To assess service climate, we used eight items adapted from Schneider and colleagues’ service climate measure (α = .91) [53]. Items were modified to apply specifically to a SUD service setting. All service climate items were scored on a 0 (“poor”) to 4 (“excellent”) scale.

#### Organizational climate

Molar organizational climate was measuring using the Organizational Climate Measure (OCM; [54]). The OCM in its entirety consists of 17 scales capturing a broad range of molar organizational climate. Consistent with the original measurement development study [43], three of the 17 scales were used to examine construct-based validity. The three scales included in this study assessed climate for performance feedback (*α* = .89, five items), involvement (*α* = .88, six items), and efficiency (*α* = .89, four items). OCM items were scored on a 0 (“not at all”) to 3 (“definitely true”) scale.

#### Organizational change

The Perceived Organizational Change (POC) measure [55] was utilized to assess organizational change. The subscales planned change (*α* = .80, three items) and psychological uncertainty (*α* = .91, four items) were used to examine construct-validity. POC items were scores on a 0 (“strongly disagree”) to 4 (“strongly agree”) scale.

### Statistical analyses

To evaluate the psychometric properties of the ICS, confirmatory factor analyses (CFA) were conducted using Mplus statistical software [56]. A confirmatory factor analysis allows researchers to test whether the proposed structure of the data (i.e., the loading of items on particular subscales or factors) aligns with the actual data collected [57]. We accounted for the nested data structure using the ‘CLUSTER’ command, and used maximum likelihood estimation with robust standard errors to adjust the standard error and chi-square values for non-normality. Although minimal, missing data were accounted for using full information maximum likelihood (FIML) estimation. In order to assess model fit, several descriptive fix indexes and recommended cutoffs were utilized, with comparative fit index (CFI) greater than .95, the root mean square error of approximation (RMSEA) less than .06, and the standardized root mean square residual (SRMR) less than .08 indicating strong model fit [58].

Cronbach’s alpha was also assessed for each of the subscales and overall ICS to evaluate internal consistency reliability. Intraclass correlations (ICC[1]s) and the average correlation within team (*a*_*wg(j)*_) for each subscale were calculated to evaluate the aggregation of the individual-level responses to the unit (i.e., team) level. Higher values on both ICC(1) and *a*_*wg(j)*_ suggest that aggregation to the unit level is appropriate. ICC(1) values ranging from .05–.20 are typically seen in applied research [59]. Three of the 65 teams were not included in the aggregation analyses as they were comprised of only one provider. Lastly, individual and team level construct-based validity of the ICS was examined by comparing the correlations with service climate, molar climate, and organizational change.

## Results

Table 2 provides the ICS scale reliabilities, item means, standard deviations, and the aggregation statistics. The ICS range of means for subscales was 1.86 to 2.63 on the 0–4 response scale, with the exception of Rewards for EBP, which was notably lower at .64. This pattern was similar to that reported by Ehrhart, Aarons, and Farahnak [43] with the biggest difference being for the Focus on EBP dimension, which had a mean of 2.63 in this sample versus 2.28 in a mental health sample.

**Table 2:** Summary statistics for the ICS total scale and subscales

	Mean	SD	α	ICC(1)	*a*_*wg(j)*_
Implementation Climate Scale total	2.04	.66	.90	.04	.76
Implementation Climate Subscales					
Focus on EBP	2.63	.88	.90	.11	.80
Educational Support for EBP	2.25	1.03	.84	.15	.77
Recognition for EBP	1.86	.97	.78	.07	.77
Rewards for EBP	.64	.88	.81	.04	.62
Selection for EBP	2.23	.97	.89	−.02	.80
Selection for Openness	2.63	.92	.85	.05	.78

Cronbach’s alpha reliabilities for the subscales and ICS total score ranged from .78–.90, demonstrating strong internal consistency reliability and in line with the findings of Ehrhart, Aarons, and Farahnak [43]. The *a*_*wg(j)*_ values for the total ICS scale were strong, ranging from .76 to .80, except for the Rewards subscale which was notable lower at .62. In order to determine the amount of dependency among observations within teams, intraclass correlations, specifically ICC(1) values, were calculated. The ICC(1) for the overall ICS scale was .04. The ICC(1) values for the subscales ranged from −.02 to .15; all values were .03 or larger except for the value for the Selection for EBP subscale. Overall, the *a*_*wg(j)*_ values were similarly strong as in the Ehrhart, Aarons, and Farahnak [43] paper, but the ICC(1) values were not as strong. Although this pattern of aggregation statistics supports the use of the ICS subscales and total scale as unit-level constructs in SUD treatment settings, it does suggest that there may be less within team variability in the implementation climate levels of SUD treatment settings relative to mental health settings.

CFA results provided support for the six-factor implementation climate model (χ^2^(120) = 324.21, *p* < 0.001; CFI = 0.92, RMSEA = 0.075, 90% CI [.066, .085], Probability RMSEA <= .000; SRMR = 0.074). Additional support for the factor structure was found as the standardized factor loadings ranged from .57 to .90 and were all statistically significant (*p*’s < 0.001), as shown in Table 3.

**Table 3:** Standardized factor loadings for the Implementation Climate Scale

ICS Factor items	6-factor Solution Factor Loadings
1. Focus on EBP	
Main goal is to use EBP effectively	.89
Think implementation is important	.89
Using EBP is a top priority	.82
2. Educational Support for EBP	
EBP trainings or in-services	.87
Conferences, workshops, or seminars	.83
Training materials, journals, etc.	.71
3. Recognition for EBP	
Held in high esteem	.86
Seen as clinical expert	.83
More likely to be promoted	.57
4. Rewards for EBP	
Financial incentives for use of EBP	.88
More likely to get a bonus/raise	.76
Accumulate compensated time	.68
5. Selection for EBP	
Previously used EBP	.84
Value EBP	.87
Formal education supporting EBP	.86
6. Selection for Openness	
Adaptable	.88
Flexible	.90
Open to new interventions	.67

Table 4 shows the correlations among the ICS subscales. In general, the correlations between the Rewards subscale and the other five dimensions (average *r* = .25) were lower than the correlations among the other five dimensions (average *r* = .45). Of particular note, the two lowest correlations were between Rewards and Selection for Openness (*r* = .16, *p* < .01) and Focus on EBP (*r* = .15, *p* < .01).

**Table 4:** Implementation Climate Scale subscale correlation matrix

	1	2	3	4	5
1. Focus on EBP	–				
2. Educational support for EBP	.56**	–			
3. Recognition for EBP	.41**	.32**	–		
4. Rewards for EBP	.15**	.26**	.44**	–	
5. Selection for EBP	.52**	.42**	.53**	.27**	–
6. Selection for Openness	.43**	.29**	.34**	.16**	.66**

Note: **p < 0.01

Correlations for the ICS total score and its six subscales with all of the proposed validity measures at the individual and team-levels can be found in Tables 5 and 6, respectively. For brevity’s sake, the primary focus was on the results for the ICS total score. As hypothesized, service climate was moderately to strongly correlated with ICS total score at both the individual (*r* = 0.57, *p* < 0.01) and team levels (*r* = 0.62, *p* < 0.01). Findings were somewhat mixed for the three dimensions of molar organizational climate relationships with EBP implementation climate. All of the dimensions had statistically significant but weak to moderate correlations with the overall ICS scale score at the individual level (performance feedback: *r* = 0.39, *p* < 0.01; involvement: *r* = 0.39, *p* < 0.01; efficiency: *r* = 0.28, *p* < 0.01). At the team level, correlations were slightly lower and the correlation with efficiency was not significant (performance feedback: *r* = 0.33, *p* < 0.01; involvement: *r* = 0.31, *p* < 0.01; efficiency: *r* = 0.23, *p* > 0.05). EBP implementation climate was positively related to perceptions of planned change (*r* = 0.30, *p* < 0.01) and negatively related to perceptions of uncertainty (*r* = − 0.24, *p* < 0.01) at the individual level, with a similar pattern at the team level (planned change: *r* = 0.38, *p* < 0.01; uncertainty: *r* = − 0.38, *p* < 0.01). Taken together, these correlations provide evidence of the construct validity of the ICS.

**Table 5:** Individual-level construct-based validity correlations of Implementation Climate Scale scores

	Focus on EBP	Educational Support	Recognition for EBP	Rewards for EBP	Selection for EBP	Selection for Openness	ICS Total
Service Climate	.42**	.43**	.33**	.24**	.45**	.50**	.57**
Organizational Climate							
Performance Feedback	.31**	.39**	.20**	.07	.27**	.37**	.39**
Involvement	.30**	.31**	.17**	.03	.34**	.48**	.39**
Efficiency	.22**	.24**	.10	.04	.25**	.30**	.28**
Perceived Organizational Change							
Planned Change	.24**	.26**	.25**	.07	.21**	.23**	.30**
Uncertainty	−.23**	−.14*	−.09	−.01	−.23**	−.30**	−.24**

Note: *N* = 326; *p < .05, **p < .01

**Table 6:** Team-level construct-based validity correlations of Implementation Climate Scale scores

	Focus on EBP	Educational Support	Recognition for EBP	Rewards for EBP	Selection for EBP	Selection for Openness	ICS Total
Service Climate	.48**	.45**	.45**	.21	.46**	.53**	.62**
Organizational Climate							
Performance Feedback	.47**	.55**	.32*	.11	.33**	.46**	.54**
Involvement	.31**	.27*	.18	−.06	.31*	.54**	.34**
Efficiency	.12	.21	.11	−.03	.23	.33*	.23
Perceived Organizational Change							
Planned Change	.39**	.27*	.36**	.001	.26*	.31*	.38**
Uncertainty	−.25*	−.28*	−.31*	−.02	−.31*	−.42**	−.38**

Note: *N* = 62; Only those teams with two or more members were included in team-level correlations; *p < .05, **p < .01

## Discussion

The goal of this study was to assess the psychometric characteristics and provide validation evidence for use of the ICS in SUD treatment settings. The ICS showed strong internal consistency reliability and acceptable fit for the overall factor structure. In addition, the pattern of correlations with other measures supported the construct validity of the ICS and were consistent our hypotheses. Taken together, these findings suggest that SUD organizations who make EBP implementation a priority also emphasize giving high quality service to clients, providing performance feedback to providers, involving employees in decision making, making sure work is done efficiently, and planning for organizational change while also reducing uncertainty around change.

Overall, the results supported the use of the ICS in SUD organizations. To date, the majority of research on EBP implementation in SUD has targeted molar organizational climate particularly through the development and use of the ORC. Although the ORC is helpful in assessing general organizational functioning [38], its application in EBP implementation remains unclear [39]. Having a brief, reliable, and valid tool to assess the extent to which the organization’s climate is supportive of EBP implementation provides more focus than the constructs assessed by the ORC. Thus, the use of the ICS allows organizations to understand the extent to which their orgnaizational environment aligns with implementation efforts, and to identify specific areas related to implementation to possibly target for leadership and organizaitonal interventions. Additionally, the availability of this measure provides researchers with a brief and pragmatic measure [60] with which to understand the factors that are related to the development of an EBP implementation climate and the organizational and implementation outcomes that result when EBP implementaiton climate levels are high [50].

The availability of a brief, and valid, tool that organizations and researchers can access to assess EBP implementation climate is particularly relevant for SUD treatment systems which have historically maintained a particularly wide science and practice gap. Very few studies on the dissemination and implementation of EBPs in SUD treatment have utilized valid measures of implementation, let alone measures of implementation climate [7]. Contributions by Damschroder and Hagedorn have recommended increased consideration of outer and inner context factors influencing EBP implementation in SUD treatment systems. Such factors include policies and incentives/rewards for implementation, engagement of implementation leaders/champions, and organizational culture for implementation [61]. Ongoing formative evaluation is critical for any implementation effort [62]. The application of a tool such as the ICS to measure EBP implementation climate in SUD treatment systems can help to guide successful EBP implementation efforts, serving to narrow this science and practice gap [5, 7, 31, 63].

Although the general pattern of results was supportive of the measure as a whole, it is notable that the findings for the Rewards dimension were not as strong as for the other dimensions. For instance, the within-unit agreement was lowest for the Rewards subscale, and the correlations between the Rewards subscale and the other dimensions were lower than the correlations among the other five dimensions. Furthermore, as shown in Tables 5 and 6, the Rewards subscale had the weakest correlations with the measures included to provide construct validity evidence, including at both the individual and team levels of analysis. These results are in line with what was found when this scale was validated in child welfare settings [51], such that the scale performed less well in these settings than in the mental health setting where the measure was originally developed [43]. There are several plausible explanations for the poorer performance of the Rewards dimension in this SUD services sample compared to mental health setting. One possibility is the generally limited availability of funds for any sort of financial reward, particularly in often underesourced SUD treatment agencies. With such a low base rate for rewards, the scale properties are affected for all subsequent analyses. In contrast, the Recognition subscale, which addresses non-financial recognition of providers for implementation efforts, performed better across most analyses. It is also possible this Rewards dimension performs more poorly in SUD services due to the fact many SUD service providers are in recovery themselves and are strongly motivated to be of service to others. A tenet of many 12-step programs often endorsed by individuals in recovery is to carry the message of the 12-steps forward to others in need. As a result, financial rewards may be interpreted as inappropriate, or incongruent, with this recovery-oriented perspective.

The question then becomes whether the scale should simply be removed for use in SUD settings. In our own work with organizations, we have found that there are creative ways to use small financial rewards to reinforce implementation efforts in a manner that is consistent with the values of SUD service providers. The inclusion of the Rewards scale could encourage management to consider what options may be available to them if implementing EBPs with fidelity is indeed a high priority for the organization, if appropriate. Further, as SUD agencies consider how best to create a positive EBP implementation climate, the base rate for the use of rewards may increase. Thus, although the ICS may perform better for research purposes without the inclusion of the Rewards subscale, there are practical reasons to use it for applied purposes.

Some limitations of the study should be noted. Although the Selection for EBP subscale generally performed well across the various analyses that were conducted, its ICC(1) value was negative, which can occur when the between group variability is smaller than the variability within groups. Given the relatively high levels of within-group agreement for this scale (based on the *a*_*wg(j)*_ values), this suggests that although individuals generally agree about this issue within their teams, the team means tended to be quite similar across the sample. A larger sample of organizations and teams would likely show more variability in whether experience with EBPs is considered in hiring systems. On a related note, because the sample came from three, relatively large SUD agencies, the findings may not be consistent with smaller agencies, particularly in more rural areas. More research with broader sampling is needed to support generalizability of the measure in these more rural SUD treatment contexts. Another limitation of the study is that the focus of the measure is EBPs in general, rather than a specific EBP. If SUD organizations are implementing multiple EBPs at any given time, the overall climate for EBP implementation is likely to be particularly relevant. In contrast, if the entire organization is focused on a single implementation, the presence of a climate for that specific EBP may emerge. Future research should consider this possibility and compare the measurement and relationships with other variables for these two different approaches to assessing implementation climate.

## Conclusions

In conclusion, the organizational context for implementation is a critical factor in setting the foundation for implementation success. Having a tool that is valid and reliable, in addition to being brief and practical for applied use, allows SUD organizations to better understand how to build a climate to support implementation and allows substance abuse researchers to better understand the role of climate in implementation effectiveness. Future research should expand on the study of this measure with additional constructs as well as establishing criterion-related validity evidence by showing its relationship with key implementation outcomes.

## Data Availability

The datasets generated and analyzed during the current study are not publicly available to protect the participants’ anonymity but are available from the corresponding author on reasonable request.

## Abbreviations

CFA: Confirmatory factor analysis
CFI: Comparative fit index
EBP: Evidence-based practice
EPIS: Exploration Preparation Implementation Sustainment
FIML: Full information maximum likelihood
ICC: Intraclass correlation coefficient
ICS: Implementation Climate Scale
NIDA: National Institute on Drug Abuse
OCM: Organizational Climate Measure
ORC: Organizational Readiness for Change
POC: Perceived Organizational Change
RMSEA: Root mean square error of approximation
SRMR: Standardized root mean square residual
SUD: Substance use disorder
